# Forecasting of the COVID-19 pandemic situation of Korea

**DOI:** 10.5808/gi.21028

**Published:** 2021-03-25

**Authors:** Taewan Goo, Catherine Apio, Gyujin Heo, Doeun Lee, Jong Hyeok Lee, Jisun Lim, Kyulhee Han, Taesung Park

**Affiliations:** 1 Interdisciplinary Program in Bioinformatics, Seoul National University, Seoul 08826, Korea; 2 Department of Statistics, Seoul National University, Seoul 08826, Korea; 3 The Research Institute of Basic Sciences, Seoul National University, Seoul 08826, Korea

**Keywords:** COVID-19, deep learning, disease transmission, mathematical model, pandemics, statistical model

## Abstract

For the novel coronavirus disease 2019 (COVID-19), predictive modeling, in the literature, uses broadly susceptible exposed infected recoverd (SEIR)/susceptible infected recoverd (SIR), agent-based, curve-fitting models. Governments and legislative bodies rely on insights from prediction models to suggest new policies and to assess the effectiveness of enforced policies. Therefore, access to accurate outbreak prediction models is essential to obtain insights into the likely spread and consequences of infectious diseases. The objective of this study is to predict the future COVID-19 situation of Korea. Here, we employed 5 models for this analysis; SEIR, local linear regression (LLR), negative binomial (NB) regression, segment Poisson, deep-learning based long short-term memory models (LSTM) and tree based gradient boosting machine (GBM). After prediction, model performance comparison was evelauated using relative mean squared errors (RMSE) for two sets of train (January 20, 2020‒December 31, 2020 and January 20, 2020‒January 31, 2021) and testing data (January 1, 2021‒February 28, 2021 and February 1, 2021‒February 28, 2021) . Except for segmented Poisson model, the other models predicted a decline in the daily confirmed cases in the country for the coming future. RMSE values’ comparison showed that LLR, GBM, SEIR, NB, and LSTM respectively, performed well in the forecasting of the pandemic situation of the country. A good understanding of the epidemic dynamics would greatly enhance the control and prevention of COVID-19 and other infectious diseases. Therefore, with increasing daily confirmed cases since this year, these results could help in the pandemic response by informing decisions about planning, resource allocation, and decision concerning social distancing policies.

## Introduction

The novel coronavirus disease 2019 (COVID-19) presents an important and urgent threat to global health. Since the outbreak in early December 2019 in the Hubei province of the People’s Republic of China, the number of patients confirmed to have the disease has exceeded 118 million as the disease spread globally, and the number of people infected is probably much higher [1]. More than 2.6 million people have died from COVID-19 (up to 11 March 2021) [2]. Despite public health responses aimed at containing the disease and delaying the spread [3,4], several countries have been confronted with a critical care crisis, and more countries could follow [5].

To mitigate and suppress the burden of COVID-19 on the healthcare system, while also protecting the general public, especially the highly susceptible group of people, robust models that predict the prognosis of COVID-19 were urgently needed to support decisions about shielding, hospital admission, treatment, and population level interventions [6]. In this situation, prediction tools can help project different scenarios such as (1) number of possible confirmed (new) cases, (2) number of possible hospitalized cases, (3) number of possible death cases and so forth. As a consequence, prediction tools are useful for several different purposes [7].

Other features, such as social distancing, stay-at-home orders, use of facemasks or self-quarantine, travel restriction, and contact tracing could help predict what comes next. For better understanding, prediction models are important for better estimation about the disease and its possible threats such as the number of cases based on the level of severity can help determine the need of numbers of ventilators and other sophisticated medical equipment. Furthermore, countries need to shape their health system responses in accordance with the need [8]. Therefore, access to accurate outbreak prediction models is essential to obtain insights into the likely spread and consequences of infectious diseases. Governments and other legislative bodies rely on insights from prediction models to suggest new policies and to assess the effectiveness of the enforced policies [9].

**Fig. 1.**
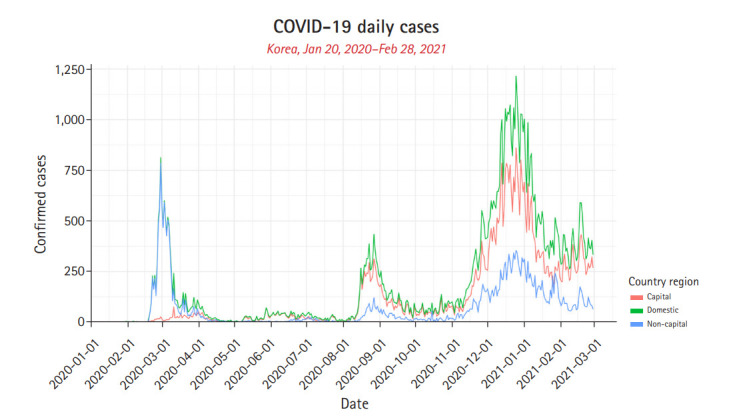
Daily confirmed cases of South Korea. Daily confirmed cases of capital, non-capital, and domestic is represented in red, blue, and green, respectively.

For COVID-19, predictive modeling, in the literature, uses broadly susceptible exposed infected recoverd (SEIR)/ susceptible infected recoverd (SIR), agent-based, curve-fitting models. Besides, machine-learning models that are built on statistical tools have widely been used too [7]. Here, we employ statistical models; segmented Poisson, negative binomial (NB), and local likelihood regression (LLR), mathematical model SEIR, deep-learning based model long short-term memory (LSTM), and tree based gradient boosting machine (GBM) for prediction of future COVID-19 pandemic situation of Korea. The COVID-19 daily confirmed cases of Korea was divided into two regions: capital area (Seoul metropolitan area) and non-capital area (non Seoul metropolitan area). Domestic which is the sum of Capital and non-capital areas was also analyzed (Fig. 1). The daily confirmed cases of these regions were then split into train (January 20, 2020‒December 31, 2020 and January 20, 2020‒January 31, 2021) and test (January 1, 2021‒Febraury 28, 2021 and Febraury 1, 2021‒February 28, 2021) datasets. The prediction performance of the above models were tested using relative mean square error (RMSE). RMSE takes the total squared error and normalizes it by dividing by the total squared error of a simple predictor. Thus, the smaller the RMSE value, the better the prediction performance of the model.

Therefore, with increasing daily confirmed cases since the beginning of 2021, in Korea and elsewhere, such models could help in the response to pandemic by informing decisions about planning, resource allocation, and decision of the social distancing.

## Methods

### COVID-19 confirmed cases data

The daily series of confirmed cases of COVID-19 for South Korea from January 20, 2020 to Febraury 28, 2021 was obtained from Kaggle (from January 20 to June 30, 2020) [10] and Korea public data portal of the Ministry of Health and Welfare (from July 1, 2020 to February 28, 2021) [11]. The combined data was divided into two regions: Capital or Seoul Metropolitan area (Capital; Seoul, Incheon, and Gyeonggi-do) and non-capital or non-Seoul Metropolitan area (non-capital; other cities beside Seoul, Incheon, and Gyeonggi-do). The analysis was conducted on the Domestic area (capital and non-capital), Seoul Metropolitan area, and non-Seoul Metropolitan area data, respectively. The data was split into two subsets. First subset is composed of training (January 20, 2020‒December 31, 2020) and test data (the last 59 days, January 1, 2021‒Febraury 28, 2021). And second subset is composed of training (January 20, 2021‒January 31, 2021) and test data (the last 28 days, February 1, 2021‒Febraury 28, 2021) for downstream analysis with the test data used for prediction analysis.

### Prediction models

As one model may not give the best prediction of the COVID-19 situation of Korea, we present prediction results estimated by different models that can apply the above data. Many models are available and have been implemented for forecasting the pandemic situation of many countries and look at afew. In this section, we introduce segmented Poisson model, LLR model, deep-learning based LSTM model, NB model, SEIR model, and GBM model used for predicting the COVID-19 situation of Korea.

### Segmented Poisson model

Here, we regarded the confirmed cases as a function of time *t* based on a segmented Poisson model. Let *Y_t_* be the confirmed cases at day *t* which is the number of days since the first case occurred. Poisson model is defined as;

$$Y_{t}\sim poisson(\mu_{t}),$$

where *μ_t_* is the expectation of *Y_t_* with segments.

Breakpoints were considered in the daily confirmed cases during the analysis by splitting the daily confirmed cases into segments (Supplemantary Table 1). These breakpoints were decided using some of the aforementioned significant events linked to the spread of COVID-19 in South Korea. Since there are three breakpoints, four segments are defined as follows:

$$\log\left(\mu_{t}\right)=\;\left\{\begin{array}{c} \;\;\;\;\;\;\;\;\;\;\;\;\;\;\;\;\;\;\;\;\;\;\;\;\;\;\;\;\;\;\;\;\;\;\;\;\;\;\;\;\;\;\;\;\;\;\;\;\;\beta_{0}+\beta_{11}t+\beta_{21}\log\left(t+1\right),\;(t=0,1,...,c_{1}-1)\\ \;\;\;\;\;\;\;\beta_{0}+\beta_{11}t+\beta_{21}\log\left(t+1\right)+\beta_{12}(t-c)+\beta_{22}\log\left(t-c+1\right),\;(t=c_{1},...,c_{2}-1)\\ \beta_{0}+\beta_{11}t+\beta_{21}\log\left(t+1\right)+\cdots +\beta_{13}(t-c)+\beta_{23}\log\left(t-c+1\right),\;(t=c_{2},...,c_{3}-1)\\ \beta_{0}+\beta_{11}t+\beta_{21}\log\left(t+1\right)+\cdots +\beta_{14}(t-c)+\beta_{24}\log\left(t-c+1\right),\;(t=c_{3},...,n) \end{array}\right.,$$

where *c_i_* (*i*=1,2,3) are breakpoints.

### NB model

NB model is defined as [12];

$$Y_{t}|F_{t-1}\sim NB(\lambda_{t},\;\phi ),$$

where *λ_t_* is the conditional expectiation of *Y_t_* given *F*_*t*-1_ as the history of the joint process {*Y_t_*,*λ_t_*:*t*∈ℕ}. Conditional mean and variance of *Y_t_* are defined as;

$$E(Y_{t}|F_{t-1})=\lambda_{t}$$

$$VAR(Y_{t}|F_{t-1})=\lambda_{t}+\lambda_{t}^{2}/\phi ,$$

where *ϕ* is the dispersion parameter. And overdispersion parameter *σ*^2^ is defined as *σ*^2^=1/*ϕ*. NBdistribution is defined as;

$$P(Y_{t}=y:|F_{t-1})=\frac{\Gamma (\phi +y)}{\Gamma (y+1)\Gamma (\phi )}{\left(\frac{\phi }{\phi +\lambda_{t}}\right)}^{\phi }\;{\left(\frac{\lambda_{t}}{\phi +\lambda_{t}}\right)}^{y}$$

where *y*=0,1,…*n*. For estimating *λ_t_*, *l*={1,7,21} were used as lagged confirmed cases and $\log\left(\lambda_{t}\right)\;=\;\beta_{0}\;+\;\sum_{i=1}^{L}\beta_{i}\;*\;\log\left(Y_{t-l_{i}}\;+1\right)$, were used for the model. For NB model, package ‘tscount’ were used to analyze the confirmed cases of South Korea as time series count data [13].

### LLR model

Our LLR model is based on Poisson model which previously mentioned. For this model, local quadratic approximation is fitted within a smoothing window of bandwidth *h*, which is the number of the nearest past observations to be used in the local fit. We use tricube kernel of weight W(u)=(1-|u|^3^)^3^ for each point. The local quadratic log-likelihood is defined as;

$$L_{t}(a)=\sum_{t=1}^{h}w_{i}(t)\;l(Y_{t},\;a_{0}+a_{1}(t_{i}-t)+a_{2}{\left(t_{i}-t\right)}^{2}),$$

where $w_{i}(t)\;=\;W(\frac{t_{i}-t}{h/2})$ and *l* are the log-likelihood function based on Poisson distribution assumption. The local likelihood estimate is made by maximizing over the parameter *a*=(*a*_0_, *a*_1_, *a*_2_)^t^.

We utilize a rolling origin cross validation to select optimal bandwidth of the smoothing window [14]. Validation sets are divided at the local peaks of counts. Validation MSE is cumulated by each validation set's counts being predicted using past validations sets. The bandwidth with the smallest validation MSE is selected as optimal bandwith. And then using optimal bandwith we finally fitted LLR model. For LLR model [15], package ‘locfit’ was used [16].

### Long short-term memory

Here, LSTM network is considered [17]. Let the input data *X_t_* be as a set of vector consisting of *Y_t-h_* to *Y_*t*-1_* according to day *t*, where *h* is the bandwidth having values {7, 14, 21, 28, 35, 42, 49, 56}. Among these, the optimal *h* is selected using validation set, which is last 7 days of the training period. The data is normalized using minmax normalizer to transform data to be in the range of 0 to 1.

**Fig. 2.**
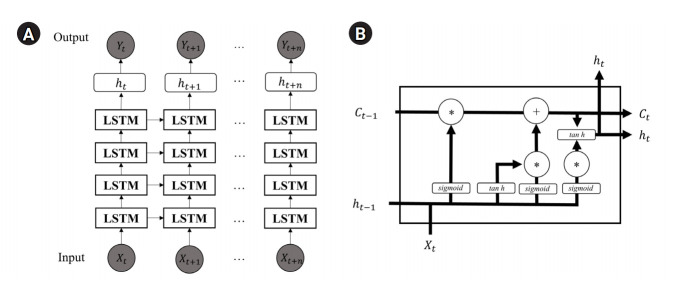
Long short-term memory (LSTM) model architecture. (A) Overall architecture of LSTM. (B) The LSTM block architecture.

The LSTM architecture is described in Fig. 2 [18]. Each blocks in the model use current input value *X_t_* with *C*_*t*-1_ and *h*_*t*-1_ to be trained. The *C*_*t*-1_ and *h*_*t*-1_ are the state and output of the last block, respectively. We assume four blocks with 64 units, each with a 0.2 dropout layer. The optimization is held using the adam optimizer to minimize the MSE during the training process.

Once the optimal model is built, it is applied to the test data for prediction. The prediction is performed sequentially by using the current output as the part of input of the next prediction. The analysis was performed using Python version 3.7.6, and ‘keras’ library.

### SEIR model with least squares

The infectious disease dynamic can be formulated with a mathematical model. We consider the SEIR model to fit the dataset of COVID-19 daily confirmed cases and predict the incidence of COVID-19 epidemic in Korea. In SEIR model, population is divided in four groups: susceptible (S), exposed (E), symptomatic and infectious (I) and recovered (R) individuals. This model includes the spread of infection during the latent period. The latency of COVID-19 infection is biologically realistic. The SEIR model is defined by the following the system of ordinary differential equation [19-22]:

$$\frac{dS}{dt}=-\beta S\frac{I}{N}\;\frac{dE}{dt}=\beta S\frac{I}{N}-\kappa E\;\frac{dI}{dt}=\kappa E-\gamma I\;\frac{dR}{dt}=\gamma I,$$

where *β* is the transmission rate, *γ* is the recovery rate, and 1/κ is the average incubation period. The initial condition of this model *S(0)*, *E(0)*, *I(0)*, *R(0)* must satisfy the condition *S(0)* + *E(0)* + *I(0)* + *R(0)* = N, where N is the total population size. In data fitting, the unknown parameters in model were estimated by a least squares algorithm. The numerical simulation and analysis were performed in MATLAB 2020a.

### Gradient boosting machine

GBM is a tree based machine learning algorithm that can be used for regression and classification problems. GBM consist of weak regression learner and decision trees. The decision tree uses the input value to determine which regression learner is best to make predictions.

Based on adaptive boosting algorithm, GBM can build a strong regression learner by iteratively combining a set of weak regression leaners. GBM use gradient descent for minimizing loss function of a strong regression learner. Like other boosting algorithms, GBM adds models into the tree using greedy style [23]:

$$F_{m}(x)=F_{m-1}(x)+\rho_{m}h_{m}(x),$$

where *F_m_* is the updated model, *F*_*m*-1_ is previous model and *ρ_m_h_m_* is the newly added model. *h_m_* is the trained base learner which minimizes the loss function L and *ρ* is the multiplier which is found by solving one dimensional optimization problem.

$$\rho_{m}=arg\;\underset{\rho }{min}\sum_{t=1}^{n}L(y_{i},\;F_{m-1}(x_{i})+\rho h_{m}(x_{i})),$$

To build GBM, ‘LightGBM’ library was used [24].

### Model assessment

To evaluate the above models, RMSEs for the train and test datasets for each of the fitted models were calculated as follows:

$$RMSE=\sum_{t=1,...,n}\frac{{\left(\hat{\mu_{t}}-y_{t}\right)}^{2}}{{\left(\hat{y}-y_{t}\right)}^{2}},$$

where *n* is the number of data points, *y_t_* is the observed values, $\hat{\mu_{t}}$ is the predicted values from a fitted model and $\bar{y}$ is the mean of observed values. To compare models predicting different regions, having different scale of confirmed cases, RMSE measure was chosen.

## Results

The COVID-19 daily confirmed cases of the country were divided into two regions (non-capital and capital) with the total being domestic and analysed using the above models. The data was split into two subsets and used in the training and prediction analysis of the models.

As for model evaluation, in Table 1, for comparison of models in the whole country and the two regions, we observe that the train RMSE is always lower than the test RMSE, with the domestic region producing the highest RMSE values for all models. Also, the segmented Poisson model gives higher RMSE values when compared with other methods. With the first data subset: in the whole country (domestic), SEIR model and GBM had the lowest train RMSE values while NB and LLR had the lowest test RMSE values. In the Capital region, GBM and LLR have the lowest train RMSE while LLR and SEIR have the lowest test RMSE values, respectively. The non-capital region showed that SEIR and GBM have the lowest train RMSE while GBM and LLR have the lowest test RMSE values, respectively.

With the second data subset: in the country, LLR and SEIR had the lowest RMSE while NB and GBM had the lowest train RMSE values, respectively. Capital region showed that LLR and NB had the lowest train RMSE while NB and GBM had the lowest test RMSE values. In the non-capital region, SEIR and LLR had the lowest train RMSE while NB and GBM had the lowest test RMSE values.

Therefore, taking into lower train and test RMSE values for all region and both data subsets, we can conclude that LLR model, GBM, SEIR model and then NB model were the best prediction models for forecasting of the COVID-19 situation of Korea. Segmented Poisson model tended to have the highest test RMSE values in all scenarios.

A look that the prediction plots of the these models shows that the daily COVID-19 confirmed cases will decline in the country (domestic), Seoul metropolitan (capital) and non-Seoul metropolitan (non-capital) areas using LLR, and NB models using the first data subset, while it will increase and stay constant using the segmented Poisson and LSTM models, respectively (Supplementary Figs. 1‒3). With the second data subset, daily COVID-19 confirmed cases will decline in the three regions as predicted by NB, segmented Poisson and LSTM models, while it will increase in the country and non metropolitan areas but will decline in the metropolitan areas, using LLR model (Supplementary Figs. 4‒6). The SEIR and GBM models shows a decrease in daily confirmed cases in the country and the two regions for all data subsets (Supplementary Figs. 7 and 8).

## Discussion

The objective of our analysis was to predict the future COVID-19 situation of South Korea using daily confirmed cases. We employed six different models in this analysis and all models gave some different prediction results for different data subsets and regions. The evaluation of the models using RMSE showed that local likelihood rgression, GBM, SEIR and NB models had the lowest RMSE values, making them the best models, though LSTM gave better RMSE values compared to segmented Poisson model. LLR, GBM, SEIR, NB and LSTM models mainly predicted a decline in COVID-19 daily confirmed cases in the country and the two regions of Korea. We can reasonably take that these results portray the future situation of the country.

With the first dataset, NB, SEIR, and LLR, respectively showed the best test performance in domestic, capital, and non-capital areas, while with second dataset, GBM showed the best test performance for all regions. In case of NB model, we found that the coefficient of confirmed cases of the day before had the largest value. This means that confirmed cases of the day before can affect the prediction of most future confirmed cases. In case of GBM, we could obtain feature importance plots of the model (Supplementary Fig. 9). We discovered that the confirmed cases of the last day was the most important feature for all regions. Thus, the models using the confirmed cases of the past days seemed to perform better than other models without using such data. The parameters of the mathematical model SEIR can be easily interpreted as a rate or transition parameter. However, the local regression does have too many parameters to assign meaningful interpretation.

Note that the number of daily confirmed cases varied across regions of Korea, so we first fitted these models for each of the regions and compared them with the number of observed cases. However, the comparison of prediction models based on regional data was not convincing due to the small sample sizes. Instead of fitting the models for each region, we considered combining the two regions of capital and non-capital areas, which provided enough sample sizes. We also tried predicitng the number of deaths due to COVID-19. However, the data was not large enough (with only 1,669 deaths as of March 14, 2021) [25] to provide reliable fitted results from the models.

In our study, there is one challenge that predictions made reflect interventions in place at the time the model was developed. So, one can argue the influence of government intervention policies in the above observed results. However, our comparison result is still valid because all models reflected the same intervention effects. Actually, the Korean government has maintained a high level of social distancing with a ban in gatherings of more than five persons [26], in their efforts to lower the triple-digit number of daily confirmed cases that has been observed since the start of this year. According to Heo et al.’s study [27] on the COVID-19 situation of Korea, the Korean government social distancing policy was predicted to lead to a decrease in the daily confirmed cases observed in the country but with only segmented Poisson model which according RMSE value, did not perform well as compared to the other models. In future, we hope to control for the influence of government interventions using the other models, to give a whole picture of the future COVID-19 situation of the country.

A good understanding of the epidemic dynamic would greatly enhance the control and prevention of COVID-19 as well as other infectious diseases. Therefore, taking precaution when using prediction to support a decision, for example, return to work or lowering of the social distancing level, is highly encouraged too.

## Figures and Tables

**Table 1.** RMSE for the regions and models following the the two data subsets

Region	Model	RMSE of data split 1		RMSE of data split 2	
		Train	Test	Train	Test
		(Jan 20, 2020‒Dec 31, 2021)	(Jan 1, 2021‒Feb 28, 2021)	(Jan 20, 2020‒Jan 31, 2021)	(Feb 1, 2021‒Feb 28, 2021)
Domestic	Segmented Poisson	0.088	1194.103	0.251	16.415
	Negative binomial	0.057	0.409	0.063	2.088
	Local regression	0.037	0.793	0.039	14.856
	LSTM	0.051	23.117	0.083	6.327
	SEIR	0.033	0.956	0.035	2.658
	GBM	0.022	1.507	0.082	0.591
Capital	Segmented Poisson	0.075	668.199	0.235	5.312
	Negative binomial	0.061	1.311	0.064	3.078
	Local regression	0.042	1.135	0.046	3.846
	LSTM	0.054	14.8	0.074	3.934
	SEIR	0.073	0.410	0.072	3.109
	GBM	0.021	1.960	0.095	0.892
Non-capital	Segmented Poisson	0.118	1131.838	0.195	34.935
	Negative binomial	0.097	0.912	0.103	1.157
	Local regression	0.074	0.522	0.076	33.232
	LSTM	0.087	15.207	0.119	4.774
	SEIR	0.036	0.610	0.036	1.964
	GBM	0.015	0.607	0.083	0.855

RMSE, relative mean squared error; LSTM, long short-term memory; SEIR, susceptible exposed infected recoverd; GBM, gradient boosting machine.

## Supplementary Materials

Supplementary Table 1. Breakpoints used for segmented Poisson model

Supplementary Fig. 1. Prediction of the coronavirus disease 2019 (COVID-19) situation for the domestic region with the first data subset. LSTM, long short-term memory.

Supplementary Fig. 2. Prediction of the coronavirus disease 2019 (COVID-19) situation for the Capital region with the first data subset. LSTM, long short-term memory.

Supplementary Fig. 3. Prediction of the coronavirus disease 2019 (COVID-19) situation for the non-capital region with the first data subset. LSTM, long short-term memory.

Supplementary Fig. 4. Prediction of the coronavirus disease 2019 (COVID-19) situation for the domestic region with the second data subset. LSTM, long short-term memory.

Supplementary Fig. 5. Prediction of the coronavirus disease 2019 (COVID-19) situation for the capital region with the second data subset. LSTM, long short-term memory.

Supplementary Fig. 6. Prediction of the coronavirus disease 2019 situation for the non-capital region with the second data subset. LSTM, long short-term memory.

Supplementary Fig. 7. Prediction of the coronavirus disease 2019 (COVID-19) situation for the three regions with the first and second data subset using susceptible exposed infected recoverd (SEIR).

Supplementary Fig. 8. Prediction of the coronavirus disease 2019 (COVID-19) situation for the three regions with the first and second data subset using gradient boosting machine (GBM).

Supplementary Fig. 9. Feature importance of the coronavirus disease 2019 situation prediction for all region with the second data.
