# Supplementary material for: Forecasting of the COVID-19 pandemic situation of Korea
Source: Genomics Inform. 2021 Mar 25;19(1):e11. doi: 10.5808/gi.21028 (PMC8042305; doi:10.5808/gi.21028)
Supplement: Supplementary Table 1. — Breakpoints used for segmented Poisson model [file gi-21028suppl1.docx]

**Supplementary Table 1.** Breakpoints used for segmented Poisson model

| Breakpoints | Date | Significant events |
| --- | --- | --- |
| 1st breakpoint | February 17, 2020 | The first case related to a church service in Daegu |
| 2nd breakpoint | August 10, 2020 | The first Monday after the peak summer vacation season |
| 3rd breakpoint | November 14, 2020 | Large-scale rallies held by the Korean Confederation of Trade Unions attended by more than 10,000 people across the country |
