# Supplementary material for: Forecasting of the COVID-19 pandemic situation of Korea
Source: Genomics Inform. 2021 Mar 25;19(1):e11. doi: 10.5808/gi.21028 (PMC8042305; doi:10.5808/gi.21028)
Supplement: Supplementary Fig. 4. — Prediction of the coronavirus disease 2019 (COVID-19) situation for the domestic region with the second data subset. LSTM, long short-term memory. [file gi-21028suppl5.docx]

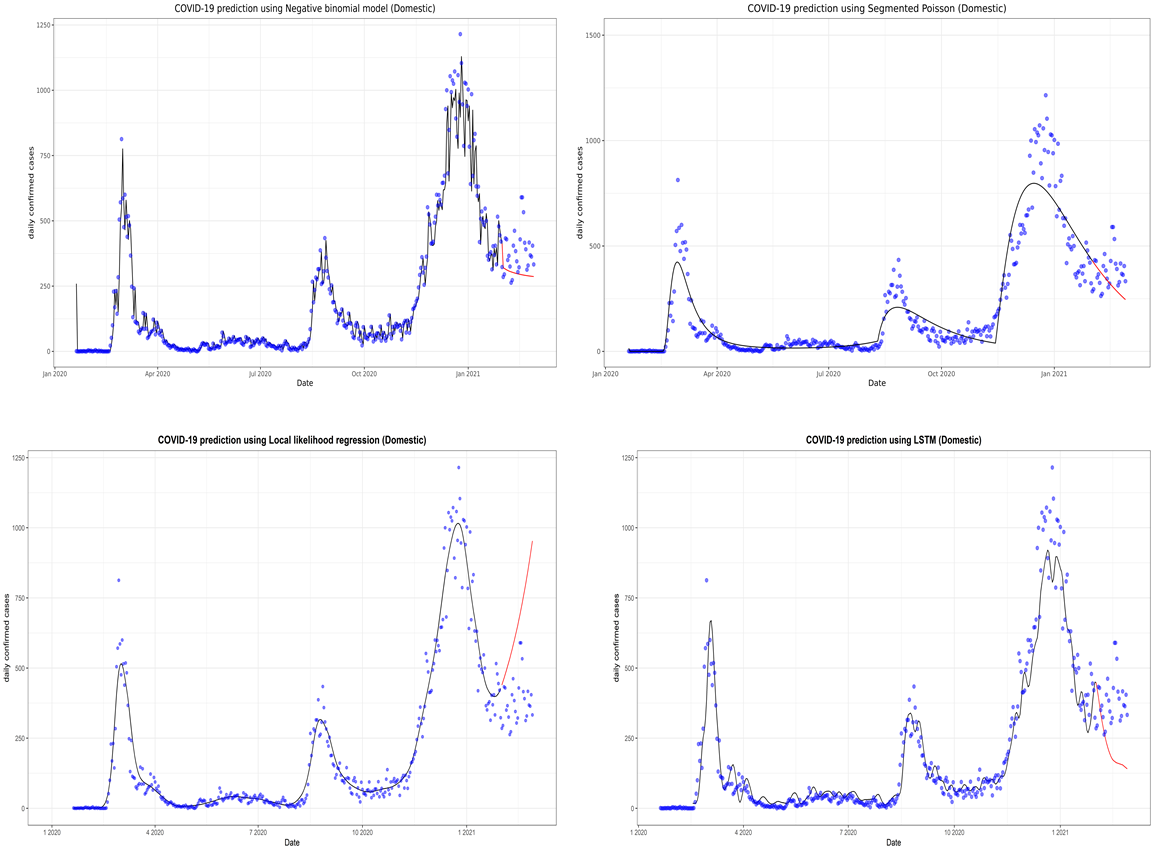


**Supplementary Fig. 4**. Prediction of the coronavirus disease 2019 (COVID-19) situation for the domestic region with the second data subset. LSTM, long short-term memory.
