# Supplementary material for: Forecasting of the COVID-19 pandemic situation of Korea
Source: Genomics Inform. 2021 Mar 25;19(1):e11. doi: 10.5808/gi.21028 (PMC8042305; doi:10.5808/gi.21028)
Supplement: Supplementary Fig. 6. — Prediction of the coronavirus disease 2019 situation for the non-capital region with the second data subset. LSTM, long short-term memory. [file gi-21028suppl7.docx]

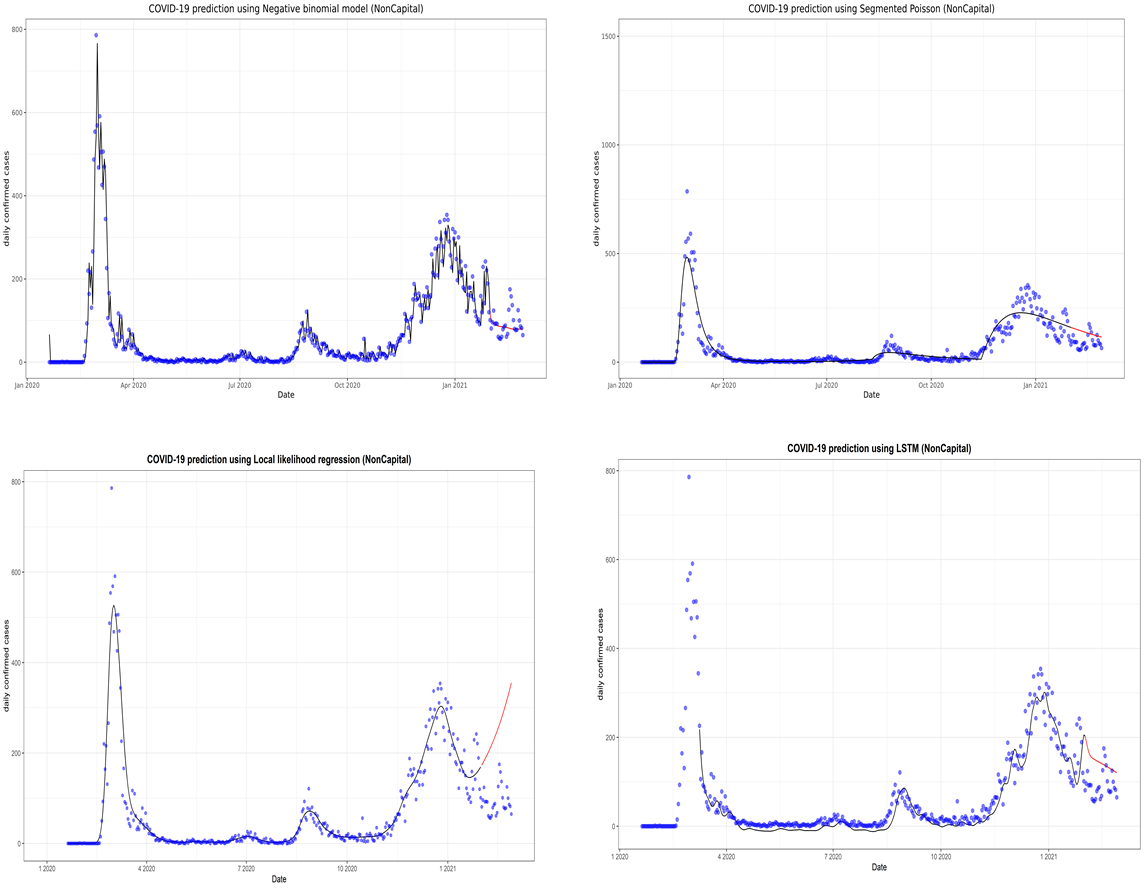


**Supplementary Fig. 6**. Prediction of the coronavirus disease 2019 situation for the non-capital region with the second data subset. LSTM, long short-term memory.
